# Unity and ROS as a Digital and Communication Layer for Digital Twin Application: Case Study of Robotic Arm in a Smart Manufacturing Cell

**DOI:** 10.3390/s24175680

**Published:** 2024-08-31

**Authors:** Maulshree Singh, Jayasekara Kapukotuwa, Eber Lawrence Souza Gouveia, Evert Fuenmayor, Yuansong Qiao, Niall Murry, Declan Devine

**Affiliations:** 1 Polymer, Recycling, Industrial, Sustainability and Manufacturing Research Institute, Athlone Campus, Technological University of Shannon: Midland and Midwest, N37 HD68 Athlone, Ireland; 2 Software Research Institute, Athlone Campus, Technological University of Shannon: Midland and Midwest, N37 HD68 Athlone, Irelandyuansong.qiao@tus.ie (Y.Q.); niall.murray@tus.ie (N.M.)

**Keywords:** digital twins, ROS, unity, MoveIt, robotics

## Abstract

A digital twin (DT) is a virtual/digital model of any physical object (physical twin), interconnected through data exchange. In the context of Industry 4.0, DTs are integral to intelligent automation driving innovation at scale by providing significant improvements in precision, flexibility, and real-time responsiveness. A critical challenge in developing DTs is achieving a model that reflects real-time conditions with precision and flexibility. This paper focuses on evaluating latency and accuracy, key metrics for assessing the efficacy of a DT, which often hinder scalability and adaptability in robotic applications. This article presents a comprehensive framework for developing DTs using Unity and Robot Operating System (ROS) as the main layers of digitalization and communication. The MoveIt package was used for motion planning and execution for the robotic arm, showcasing the framework’s versatility independent of proprietary constraints. Leveraging the versatility and open-source nature of these tools, the framework ensures interoperability, adaptability, and scalability, crucial for modern smart manufacturing applications. Our approach was validated by conducting extensive accuracy and latency tests. We measured latency by timestamping messages exchanged between the physical and digital twin, achieving a latency of 77.67 ms. Accuracy was assessed by comparing the joint positions of the DT and the physical robotic arm over multiple cycles, resulting in an accuracy rate of 99.99%. The results highlight the potential of DTs in enhancing operational efficiency and decision-making in manufacturing environments.

## 1. Introduction

Digital twins (DTs) are becoming increasingly important across a variety of industries for the virtual mapping of physical systems and processes. A digital twin (DT) is a virtual replica that mimics its physical twin in real time, and vice versa [1]. This continuous data exchange differentiates DTs from digital shadows, which only reflect changes after the fact, lacking the immediate feedback loop essential for dynamic and adaptive systems [2]. DTs are used in manufacturing to simulate production processes and optimize efficiency, in aerospace to simulate aircraft behaviour and predict performance, in construction to optimize building design and construction processes, and in healthcare to simulate and predict the behaviour of biological systems. The wide range of DT applications showcases its versatility and transformative potential, making it a powerful tool for optimization, prediction, and decision-making in various industries [3].

Robotics in manufacturing are well-known for enhancing productivity and product quality due to their durability, accuracy, and flexibility. DTs can describe, control, and display robotic systems’ behaviour in real time, enabling the intelligent perception, simulation, understanding, prediction, and optimization of manufacturing processes [4,5]. This supports developing control strategies to boost productivity and product quality while reducing costs [6,7,8]. Moreover, by simulating real-world conditions, DT allows for extensive testing of the equipment and operators’ training without the risks associated with physical prototypes, such as damage to the equipment or safety hazards to staff [3]. DT also minimizes the environmental impact of manufacturing processes by optimizing resource utilization and reducing waste [9]. Applied across all production stages—from design and prototyping to testing and maintenance—DTs, integrated with IoT, AI, and big data analysis, are revolutionizing product design, manufacturing, and servicing [10,11]. This holistic approach is pivotal in industries ranging from healthcare, where robotic arms are used for remote surgery [12], to construction, agriculture, and the military [13,14].

To harness these benefits, robust digital and communication layers are required. Such layers enable effective communication and collaboration between the physical and digital worlds. The use of DTs in robotics is also becoming more accessible to small- and medium-sized businesses, as a result of the availability of open-source platforms and affordable hardware [15]. Unity is a game engine technology that can be applied to create 3D digital representations of physical systems. It supports the creation of complex and dynamic models that faithfully replicate real-world elements in a virtual environment, making it popular within research for DT development [16]. Conversely, Robot Operating System (ROS), an open-source framework for robot software development, stands out as a powerful middleware that simplifies the communication between a physical and digital counterpart. ROS provides a collection of tools, libraries, and conventions that simplifies the task of creating complex and sophisticated robot behaviours [17]. The integration of Unity and ROS in the realm of DT technology represents a significant leap in bridging the gap between virtual simulations and physical robotics.

Despite significant advancements in DT technology, notable limitations and gaps remain in the current research. A major challenge is the scalability of DTs, especially in large and complex industrial settings where real-time data integration from multiple sources is necessary. In this work, we evaluate the proposed Unity/ROS framework as a conduit for real-time data exchange, addressing a well-known and challenging problem [3,18]. This paper aims to address some of these limitations by presenting the potential of Unity and ROS as foundational layers for digitalization and communication in the development of DTs. Additionally, while many studies concentrate on specific case studies or applications, there is a shortage of comprehensive frameworks that can be broadly applied across various industries. The methodology adopted in this study is centred on a case study of a robotic arm in a smart manufacturing cell, demonstrating the practical application and efficacy of our approach, which can be adapted for any industry using robotic arms. Moreover, while many studies discuss the implementation of Unity and ROS for DT development, there is a scarcity of detailed technical assessments regarding their real-time performance. Our study thoroughly evaluates and analyzes the latency and accuracy of the system, highlighting the framework’s efficiency and reliability. The accuracy tests assess how accurately DT mirrors the movements and positions of the physical robotic arm by comparing their joint positions over time. On the other hand, the latency tests measure the delay between commands given to DT and the corresponding actions carried out by the physical robotic arm. The proposed framework is designed to be flexible and scalable, facilitating the seamless adaptation to different robotic systems and industrial scenarios. By bridging the digital and physical worlds seamlessly, this paper contributes to the broader discourse on intelligent automation and the future of robotics in various industries.

### 1.1. Related Work

The development of DTs for robotic arms using ROS has become increasingly vital for enhancing simulation capabilities, real-time monitoring, and control accuracy in the robotics industry. S. Baidya et al. [19] created an ROS-based DT of the Franka Emika Panda robot using the Gazebo simulator, emphasizing autonomous and remote operations to manage environmental uncertainties and ensure safety. N. Kousi et al. [20] developed the DT of an assembly line employing mobile dual-arm robots that move throughout the factory, multitasking as well as assisting people, thereby improving the overall efficiency of the factory. G. Garg et al. [21] also built the ROS-based DT of a Fanuc robot which can be used for trajectory programming for complex edges and space-constrained environments, and gathering synthetic data for machine-learning applications. T.I. Erdei, R. Krakó, and G. Husi [22] developed the DT of a training centre for an industrial robotic arm, KUKA KR5, for educational purposes. Their aim was to provide training to the students using this DT of this industrial lab in a safe environment before they start the actual physical training to reduce the risk of accidents while also reducing the costs associated with physical installations and maintenance. Similarly, Z. Wang et al. [23] developed a bidirectional linkage robot DT system for educational purposes based on ROS, enhancing the real-time monitoring and control of the robot. For teleoperation and visualization purposes, D. Diachenko et al. [24] developed a DT of the OMRON TM5-900 collaborative industrial robot, highlighting the use of the MQTT protocol for synchronization and advanced user interfaces. N. Kousi et al. [25] also previously created a DT for reconfigurable assembly systems to integrate human–robot collaboration to enhance system flexibility and adaptability in production lines in the automotive sector. While significant progress has been made in developing DTs for robotic arms, a comprehensive solution integrating various capabilities remains elusive. Each study contributes uniquely, but challenges such as complexity in integration, and specific application limitations persist. Additionally, except for G. Garg et al. [21], none of the aforementioned works of research had tested their DT performance. The contributions of ROS-based DT developed by researchers is summarized in Table 1.

Currently, researchers are also combining DTs with other technologies such as virtual reality (VR), machine learning (ML), and reinforcement learning (RL) to enhance their functionality and applicability. V. Havard et al. [26] proposed an architecture for the co-simulation and communication between DTs and VR software for human–robot collaborative workplace design and assessment. By integrating DTs and VR, they created a more realistic and immersive environment for designing and testing collaborative robot workspaces in contrast to the traditional methods, which often rely on static models or less interactive simulations, lacking dynamic real-time interaction.

A VR-based DT for multi-robot systems was developed by L. Pérez et al. [27], which could be used for operator training, real-time monitoring, and feasibility studies of future optimizations. It also allowed for a more interactive and dynamic training environment for robot operators, improving their skills and performance. Researchers are also integrating DTs of robots with RL to virtually train a robotic arm to complete a given task [28,29,30]. The use of RL with DTs allows for the more effective training of robots and enables them to adapt to changing environments and tasks. In addition, using ML with DTs, researchers have been able to train industrial robots for adaptive path planning to bypass objects and people in the workspace, making it safer for human operators [31]. Overall, combining DTs with VR, RL, and ML opens new avenues for research and development in the field of robotics, enabling researchers to create more advanced and efficient robotic systems that can adapt to changing environments and tasks. The landscape of the related literature burgeons with instances where the integration of game engines with ROS has propelled DT creation to new heights, thereby underpinning the essence of our architectural choices.

The field of DT in robotics has seen significant contributions, particularly in the integration of game engines and simulation platforms. While the use of ROS and game engines like Gazebo and Unity is not novel, our approach to integrate Unity with ROS is unique in terms of targeting enhanced real-time performance and high accuracy in smart manufacturing applications. Importantly, the accessibility and open-source nature of these tools, especially ROS and Unity (for students and researchers), play a crucial role in our approach. By leveraging open-source software, we can ensure that our solutions are widely accessible to the research community and industry practitioners, fostering innovation and collaboration. Our focus is on combining Unity and ROS for advanced scalability and flexibility, in addition to the latency and accuracy analysis. As a result, it has many applications in line with Industry 4.0 and 5.0. Our work, thus, not only builds upon but also significantly expands the scope of DT applications in the realm of intelligent automation.

### 1.2. Game Engines for DT

Game engines are traditionally used by software developers to design video games, providing developers with powerful tools for creating highly immersive and realistic experiences. They are excellent software for accurately simulating light reflection, particles, gravity, and other real-world aspects [32]. However, game engines are not just limited to the gaming industry, and their advanced features have opened new possibilities for creating DTs of real-world objects and systems [33]. Owing to the rapid development in game engines, their functionalities of visualizing, training, and testing have opened them up for application in creating DTs of several things such as vehicles [34,35], robotics/manufacturing [21,29,36], smart cities [37,38], etc. to optimize operations, reduce costs, and improve safety.

**Figure 1:**
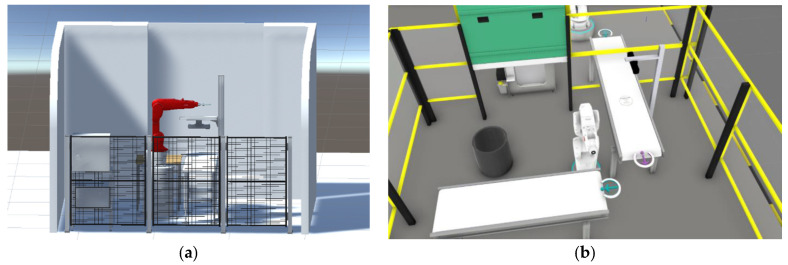
The contrast between the two-simulations graphics in Unity (**a**) vs. Visual Components (**b**).

Game engines allow us to accurately create DTs that simulate complex systems and environments (Figure 1). Unity, as one game engine example, provides a realistic and immersive environment for visualizing and interacting with the digital world. Its robust physics engine enables accurate collision detection and realistic object behaviour, enabling researchers to assess the feasibility and safety of various manufacturing processes [39]. Another recently emerging possibility with game engine technology is their ability to generate synthetic data in a shorter time, which can be used to train AI models. This is particularly useful in industries such as autonomous vehicles and robotics, where AI is being used to automate processes and improve efficiency. Unity is one of the most popular game engines with a large community of developers and a wide range of features and capabilities. The benefits of using Unity, in particular, include flexibility, ease of use, and extensive community support.

Besides all the aforementioned benefits, another reason for choosing Unity for creating the digital model is its compatibility with ROS. ROS facilitates seamless communication between digital simulations and physical objects. It enables the real-time transmission of control commands and sensor data to improve simulation accuracy [40]. In this work, a case study of a robotic arm DT using Unity and ROS as the digital and communication layers, respectively, has been developed as part of an intelligent manufacturing cell. The integration of Unity with ROS enhances its flexibility in adoption, regardless of the type of robotic arm used. This integration not only demonstrates the practical applications of DTs but also highlights the potential for future advancements in smart manufacturing technologies.

## 2. Methodology

The setup of DT comprises three distinct but interconnected layers: physical, digital, and communication layer. This section provides an overview of these layers followed by the system architecture. This architecture is not just a framework for combining different layers but a designed system that ensures dynamic, real-time interaction and adaptation of the DT based on continuous data feedback.

### 2.1. Setup

#### 2.1.1. Physical Layer

The physical setup of the manufacturing cell or pilot line has been described in detail in [41] for the interested reader. The two primary components are: the physical robot; and the digital model created in Unity. The robotic arm used for the experimentation is ABB IRB 1200-5/0.9 (ABB Ltd., Warrington, UK). The specifications and other information regarding the working range and maximum speed of each axis of the robotic arm are listed in Table 2. These specifications dictate the performance and capability of the robotic arm. The IRC5 compact controller, developed by ABB, controls the I/O ports and connects the robotic via Local Area Network (LAN) port, allowing its connection to ROS scripts. The controller utilizes the EtherNet/IP communication protocol, which operates over IEEE 802.3 for real-time communication between industrial devices.

The operational environment for the robotic arm is a smart manufacturing cell designed to replicate typical conditions in modern industrial settings. The manufacturing cell is configured to handle tasks that require high precision and flexibility, such as assembling small components, manipulating delicate materials, and coordinating with other automated systems. The robotic arm operates within a predefined workspace equipped with safety barriers and monitoring systems to ensure safe interaction between the robotic arm and human operators. The physical setup not only provides a realistic environment for testing the DT but also ensures that the findings are applicable to a wide range of industrial contexts, thereby enhancing the study’s relevance and impact.

#### 2.1.2. Digital Layer

A digital model of the robotic arm was developed in Unity. The digital model replicates the physical robot’s movements and interactions with high accuracy, enabling comprehensive simulation and analysis. In the study Unity version 2022.3 was used for creating DT. There are some prerequisites for Unity to be imported, in order to create the simulation. The packages used are listed in Table 3. These packages facilitate the integration of Universal Robot Description Format (URDF) files and enable connection between Unity and ROS. All these packages are by Unity technologies and are available on GitHub [43]. The simulation created in Unity was controlled by ROS. ROS-Unity communication can be implemented using various methods, namely, ROS#, ROS-TCP-Connector, and ROS.NET. Among these, ROS-TCP-Connector is recommended due to its superior performance, exhibiting the lowest latency and highest publishing rates across most data sizes [44].

#### 2.1.3. Communication Layer

Robot Operating System (ROS) serves as a communication layer for the creation of a DT. ROS is an open-source framework widely adopted in the robotics community due to its versatility and extensive support for various hardware and software platforms [45]. In this study, we used ROS Noetic, the latest long-term support release, which provides improved stability and support for Ubuntu 20.04. ROS provides a robust and flexible framework that enables seamless communication and data exchange between different components of the DT, including the virtual manufacturing cell and physical robot. By leveraging ROS’s publisher–subscriber architecture, a reliable and efficient communication channel can be established, facilitating the exchange of sensor data, control commands, and state information in real time [40].

In the publisher–subscriber model, the publisher node sends out information/messages to a topic, while the subscriber node receives this information by subscribing to the same topic. The nodes operate independently without any direct knowledge of one another, interacting exclusively through the exchange of messages on agreed-upon topics. This approach allows for loose coupling and high flexibility [46]. This setup ensures synchronization of the virtual and physical environments, ensuring that the DT accurately reflects the behaviour and performance of the manufacturing cell. ROS owing to its modular design also allows developers to easily integrate and customize different nodes and packages to meet specific application requirements. In addition, ROS’s extensive library of built-in communication protocols and message types simplifies the implementation of data exchange, enabling researchers to focus on developing high-level control algorithms and simulation scenarios.

The use of ROS as a communication layer enhances the fidelity and interactivity of the DT, enabling detailed analysis and optimization of manufacturing processes with a higher degree of accuracy and realism [47]. Furthermore, ROS’s active community and extensive documentation provide valuable resources for troubleshooting and continuous improvement, making it a preferred choice for developing advanced robotic applications.

### 2.2. Creation of DT

**Figure 2:**
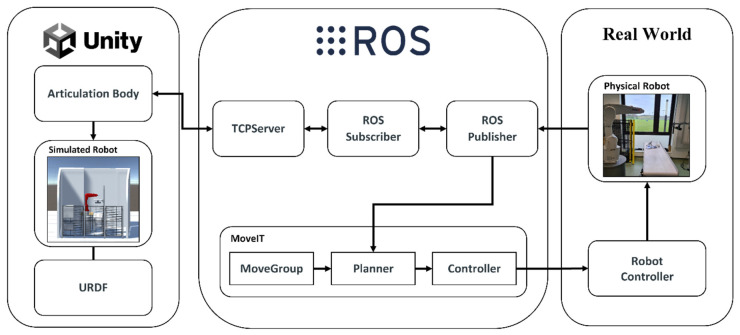
Illustration of the DT system using ROS and Unity; includes physical hardware, ROS communication, and Unity simulation layers for real-time data exchange and synchronization.

The architecture presented in Figure 2 was designed to ensure high fidelity and real-time synchronization between the physical twin and its DT. The architecture consists of several key components:

- Robot Controller: This manages the physical movements of the robotic arm, ensuring precise execution of tasks. It interacts with the actuators and sensors to implement control algorithms developed in MoveIt 1 - Noetic.
- ROS Publisher/Subscriber: The ROS Publisher broadcasts critical data such as status updates, sensor readings, and commands. The ROS Subscriber listens for incoming messages, including simulation results and new instructions for the DT, maintaining a continuous feedback loop.
- TCP Server: The Transmission Control Protocol (TCP) Server facilitates a Transmission Control Protocol/Internet Protocol (TCP/IP)-based communication protocol for the exchange of information between ROS and Unity. This server ensures reliable and efficient data transfer, minimizing latency and maximizing synchronization accuracy.
- Unity’s Articulation Body: This acts as the digital counterpart of the physical robot, where the robot’s behaviours and potential movements are simulated with high fidelity by subscribing to the ROS messages. It leverages URDF to accurately replicate the robot’s physical properties and motion dynamics within the simulation environment. The articulation body ensures that the DT behaves in a manner that is consistent with the physical robot, providing a realistic simulation environment.
- MoveIt: Within ROS, MoveIt orchestrates complex maneuvers by enabling collective planning and movement control of multiple robotic joints. MoveIt’s advanced algorithms allow for precise motion planning, collision avoidance, and task execution, which are critical for complex robotic operations.

This integrated architecture empowers the DT system to not only mirror the physical robotic arm in real time but also employ predictive and adaptive strategies that refine the robot’s operations. Such a system is invaluable in smart manufacturing settings where the DT can simulate, optimize, and improve the robotic arm’s functions in real time, ensuring maximum efficiency, safety, and productivity. The continuous data feedback loop between the physical twin and DT enables ongoing performance improvements and operational insights.

#### 2.2.1. Importing URDF to Unity

Universal Robot Description Format or URDF is a specification to describe a robot in ROS based on Extensible Markup Language (XML) [48]. It provides information about the kinematic and dynamic description of the robot, visual representation of the robot, and collision model of the robot. The description of a robot consists of a set of link elements, which represent the physical components of the robot, and a set of joint elements that connect the links and define their relative motion.

The URDF-Importer package allows developers to import a URDF file directly into Unity. This import process ensures that all the physical attributes and constraints of the robot are accurately represented in the digital model, providing a high-fidelity simulation environment. This enables the simulation of all the links and joints of the robot within the Unity environment. Both Unity and ROS share the same copy of the robot descriptor URDF, ensuring that the simulated robot in Unity accurately reflects the physical robot being controlled by ROS. In this setup, Unity serves as the main source of information regarding the joint state of the robot, allowing developers to test and refine their control algorithms in a virtual environment before deploying them on the physical robot

#### 2.2.2. Integration of Unity and ROS

To enable communication between Unity and ROS, a TCP endpoint is used to handle all messages passing between the two systems. This endpoint runs as an ROS node and provides the necessary functions for publishing, subscribing, and calling services on the ROS side. On the Unity side, an ROSConnection component is used to interface with the TCP endpoint and manage communication with ROS.

For messages to be correctly passed between Unity and ROS, they must be serialized in the same way as ROS serializes them internally. This is achieved using the MessageGeneration plugin, which generates C# classes from ROS messages. These classes include serialization and deserialization functions that allow messages to be correctly encoded and decoded when being passed between Unity and ROS [49].

**Figure 3:**
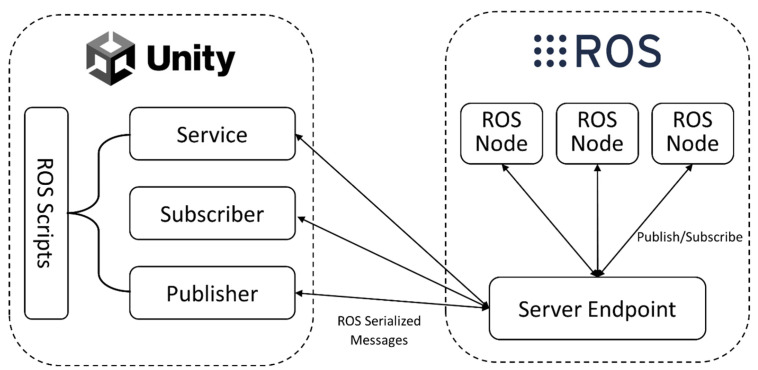
Illustration of server Endpoint sending and receiving messages between ROS nodes and Unity (adapted from [50]).

By using this architecture, developers can integrate Unity and ROS (Figure 3), allowing rapid development and testing of complex robot behaviours in a virtual environment before deploying them on physical hardware.

#### 2.2.3. MoveIt for Motion-Planning

MoveIt is a powerful motion-planning library for the ROS developed by D. Coleman et al. [51]. It is an open-source software framework that can be used in a wide range of robotic applications, including manufacturing, logistics, and service robotics. MoveIt provides a comprehensive suite of tools for motion planning, collision checking, kinematics, and control, making it an indispensable component of our DT architecture. MoveIt has been designed to be modular, extensible, and easy to use, allowing developers to quickly and efficiently develop motion-planning and control applications for robots. A key feature of MoveIt is its ability to generate pick-and-place motions for different object types. This allows robots to perform a wide range of tasks such as assembling parts, packing boxes, or handling delicate objects. MoveIt also includes tools for calculating the forward and inverse kinematics of a robot, which are essential for accurately controlling its movements. For our study, MoveIt 1 - Noetic was used for used.

In addition to its motion-planning capabilities, MoveIt provides extensive functionality for planning collision-aware trajectories. This ensures that the robot can move safely and efficiently within its environment without colliding with obstacles or other objects. MoveIt also integrates with Unity, allowing developers to use the information streamed from the Unity simulation to plan the robot’s route and send the arm path back to Unity for manipulation of the joints [49]. Overall, MoveIt is a powerful tool for developing advanced motion-planning and control applications for robots. Its modular design and extensive functionality make it an ideal choice for developers working on a wide range of robotic projects, ensuring flexibility and scalability.

Integrating Unity with MoveIt allows us to take advantage of the advanced simulation capabilities of Unity and the powerful motion-planning tools provided by MoveIt. To integrate Unity with MoveIt, the ROS-TCP-Connector package has been used. This allows Unity to send information about the simulated robot and its environment to MoveIt, which can then be used to plan collision-free trajectories for the robot. Once a trajectory has been planned, MoveIt can send the arm path back to Unity, where it can be used to manipulate the joints of the simulated robot.

## 3. Experimentation and Results

Latency and accuracy tests were conducted on the developed DT to analyze its responsiveness and feasibility. These tests are critical for validating the DT’s performance in replicating real-world operations. The latency analysis underscores the real-world applicability of the DT, shedding light on potential avenues for reducing communication delays. Meanwhile, a thorough accuracy assessment through statistical and graphical analyses elucidates the correlation between the joint positions and movements of the DT and the physical robotic arm over time. The results from these tests are essential for identifying potential improvements and ensuring that the DT can be reliably used in various industrial applications.

### 3.1. Latency

**Figure 4:**
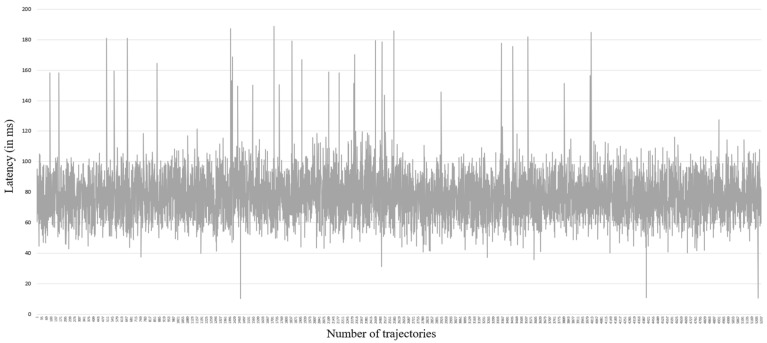
DT latency over 5000+ trajectory (X-axis: number of trajectories; Y-axis: latency in milliseconds).

Calculating the latency between a physical robotic arm and its DT involves measuring the time delay in the communication pipeline. This was achieved using timestamping messages sent and received between the physical robotic arm and the DT, and then computing the time difference. Over 5000 trajectory points were evaluated to provide a robust dataset for latency analysis. Latency data were used to calculate the mean latency and standard deviation, thereby assessing the system’s stability and performance variability. The results are presented in Figure 4. They show the time delay (in milliseconds) between command issuance and execution, capturing the lag of the DT in replicating the physical robotic arm’s operations. The DT exhibited a mean latency of 77.67 ms ± 15.74 ms. Low latency levels are essential for real-time applications in smart manufacturing environments. The standard deviation of 15.74 ms indicates a moderate level of variability in the system’s latency. This variability can be a critical factor for applications that demand uniform response times. Although the average latency suggests that the system is relatively swift, the range of latency values highlighted by the standard deviation underscores areas for further refinement and improvement.

The analysis of latency measurements suggests a stable performance. However, there are sporadic spikes that indicate infrequent delays in communication. These deviations may be attributed to a myriad of factors, such as network congestion or processing delays, which provide a focal point for further potential optimization. The system was connected to a local area network (LAN) with a high bandwidth and low latency, but occasional spikes were still observed. Understanding the root causes of these spikes can help in developing strategies to mitigate them, such as optimizing network configurations or enhancing processing efficiency. The ability of the DT to maintain a relatively low and stable latency highlights the efficiency of Unity and ROS integration for real-time control and feedback, ensuring that the DT can be reliably used in continuous and demanding industrial processes, enhancing both productivity and safety.

### 3.2. Accuracy

**Figure 5:**
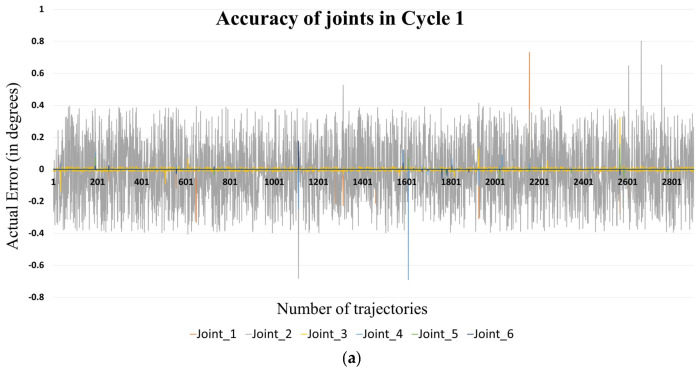

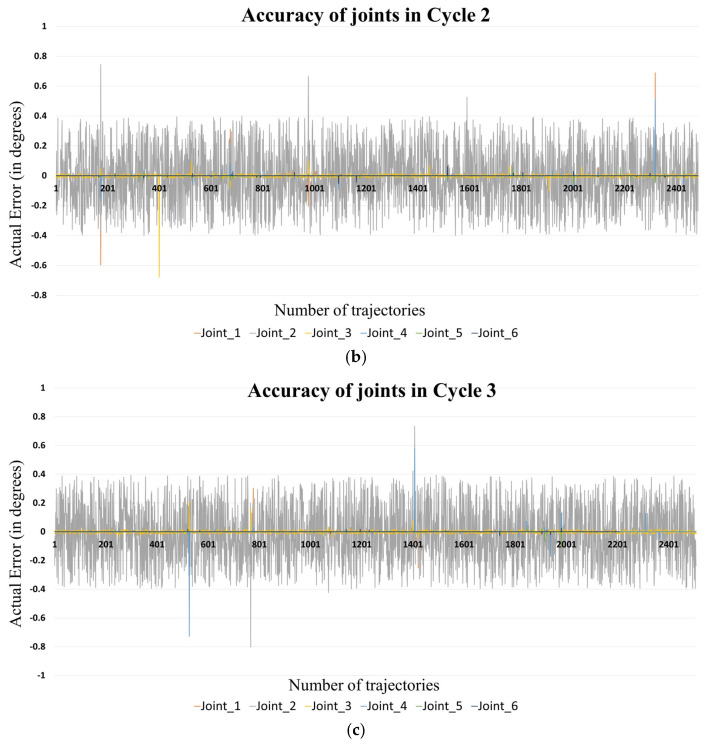
Actual error of joints in Cycle (**a**) 1, (**b**) 2, and (**c**) 3 as indicated (X-axis: number of trajectories; Y-axis: actual error in degrees).

To calculate the accuracy, the joint movement data of the DT and actual robotic arm need to be compared. It involves the comparing of the joint positions of the two systems and computing the error or difference between them. This comparison is critical for assessing how well the DT replicates the physical robot’s movements and for identifying any discrepancies that could affect performance. To minimize bias and ensure the precise measurement of accuracy, the tests were conducted for an extended duration over multiple cycles. Each cycle consisted of a randomized set of movements and joint positions, generated by a predefined algorithm, designed to simulate a variety of operational scenarios and ensure a diverse range of actions that the robotic arm might encounter in actual use. To ensure that tests reflect real-world industrial conditions, each cycle was carried out continuously over an eight-hour period, which is representative of a typical work shift. To validate the consistency of these results, the procedure was repeated across three distinct test cycles. Long-duration testing helps in capturing a comprehensive set of data, reflecting various operational conditions and potential anomalies. During each cycle, all six joint angles published by both the physical and digital robots on different ROS topics were captured in real time. The ROS ‘rosbag’ tool was used to record the data streams, ensuring that the joint angles for each robotic arm were accurately logged without any loss of information. The joint angle of the real robotic arm was then compared against the digital one. The data collected from these cycles are illustrated in Figure 5, providing a comprehensive view of the DT’s performance in terms of accuracy across repeated operations.

Both actual errors and normalized errors were calculated to provide a comprehensive understanding of the DT’s accuracy, along with their standard deviation, which was calculated to understand the variability in the accuracy measurement across different joints.

The normalized error (Equation (1)) provides a scale-independent measure of error, making it possible to compare errors across different joints having varying ranges of motion. This approach ensures that the focus is on the proportional significance of an error rather than its actual magnitude, which is crucial in applications where the impact of an error is more about its relative size compared to the operational range. For instance, a small angular error in a joint with a limited range of motion could be more critical than the same error in a joint with a much larger range. By normalizing errors, they are expressed in terms of the total possible movement range, offering a more intuitive and standardized way of understanding their significance. It also ensures that the focus is on the proportional significance of an error rather than its actual magnitude, which is crucial in applications where the impact of an error is more about its relative size compared to the operational range, rather than the actual size itself.

(1) $$\mathrm{Normalised}\;\mathrm{Error}=\frac{Actual\;Error}{Range\;of\;motion}$$

**Figure 6:**
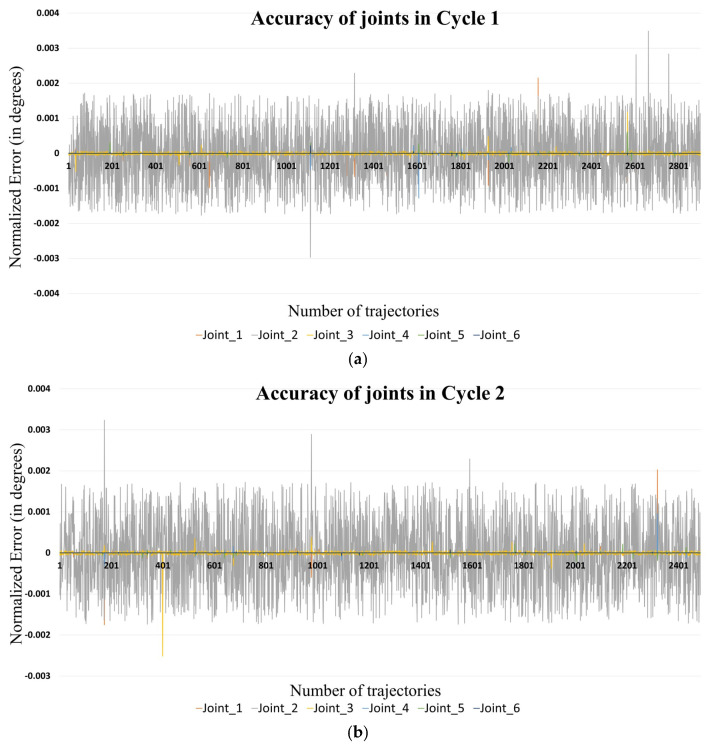

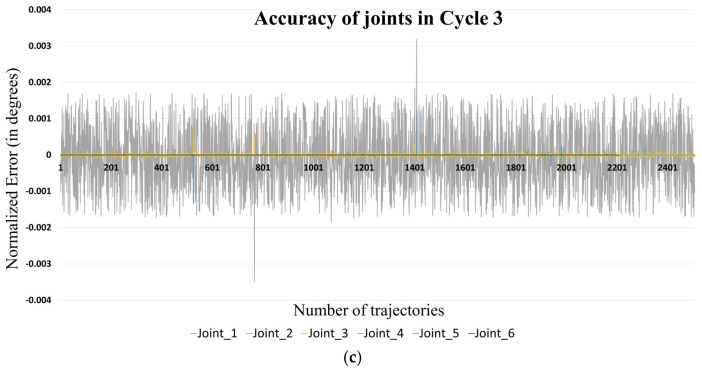
Normalized error of joints in Cycle (**a**) 1, (**b**) 2, and (**c**) 3 as indicated (X-axis: number of trajectories; Y-axis: normalized error in degrees).

The range of motion of all the joints has already been listed in Table 2. Figure 6 shows the normalized errors of all the joints at different cycles. The average/mean of absolute values of actual errors (MAE) and normalized errors (MNE) for each cycle are presented in Table 4 along with the overall average for all joints. Table 5 presents the standard deviation of actual errors (SDAE) and normalized errors (SDNE) for all the joints in each cycle as well as all cycles combined.

The accuracy for each joint was calculated using Equation (2). Although the working range of the joints is large, due to the cyclical nature of the angles, the maximum error that could be determined without an additional rotational context would be 180°. Thus, the accuracy of each joint based on the MAE is 99.99%, except for Joint_2, which has an accuracy of 99.91%, and, for the NME, it is 99.99–100%. Thus, the overall accuracy of the DT (Equation (3)) is also 99.99%. Such high levels of accuracy are critical for ensuring that the DT can be relied upon for precise control and simulation in real-world applications.

(2) $$\mathrm{Accuracy}=\left(1-\frac{\mathrm{Mean}\;\mathrm{Error}}{\mathrm{Maximum}\;\mathrm{Possible}\;\mathrm{Error}}\right)\times 100$$

(3) $$\mathrm{Overall}\;\mathrm{accuracy}=\frac{\sum \mathrm{Accuracy}}{6}$$

The standard deviation in the accuracy measurements for each joint of the DT reveals significant insights into the system’s performance. Specifically, the pooled standard deviation for the normalized accuracy data is approximately 0.000208, which is notably low. This minimal variation indicates that the accuracy of the DT is remarkably consistent across different joints, highlighting its consistency and precision in mirroring the physical robot.

The precision of the DT’s joint movements, as indicated by the high accuracy percentages, reaffirms its reliability for simulating the physical robot’s functions. Notably, Joint_2 displays a slightly lower accuracy than the others, which could be attributed either to the mechanical fault in the physical robotic arm itself or its URDF file which is simulating Joint_2 not accurately. This observation is valuable for identifying which joints or movements might require closer attention or recalibration in the physical robotic arm to ensure optimal performance. Addressing these discrepancies can further enhance the DT’s fidelity and its applicability in precision-critical tasks.

Moreover, the near-perfect normalized error metrics underscore the DT’s capability to replicate the robot’s movements within a margin that is functionally negligible, which is particularly advantageous in precision-critical applications. Such precision ensures that the DT can be used for tasks requiring high accuracy, such as assembly operations, quality inspections, and complex manipulations.

Nonetheless, it is crucial to acknowledge that, despite the accuracy observed, minor deviations can lead to significant impacts over an extended period or complex tasks. Consequently, the continuous monitoring and adjustment of the DT may be necessary to maintain this level of accuracy, ensuring that it remains a reliable tool for predicting and improving the performance of the physical robot. Regular calibration and updates to the DT based on real-world performance data can help in maintaining its accuracy and effectiveness.

## 4. Discussion

As we journey towards Industry 5.0, the demand for customization over mass production is increasing, which can be achieved by intelligent systems such as DTs which can increase productivity and competitiveness in the market. The DT is the most consistent and upward trend in the domain of industrial robotics [52]. They enable more flexible and adaptable manufacturing processes, which are essential for meeting the diverse and changing needs of modern consumers. Game engines have expanded beyond their traditional use in the gaming industry and are now being used to create DTs for a wide range of applications [33]. With their advanced features and capabilities, game engines such as Unity are becoming essential tools for industries seeking to optimize their operations and improve efficiency. The integration of game engines with robotic systems provides a highly interactive and visually rich platform for simulation, testing, and development, which significantly reduces the time and cost associated with physical prototyping. With the increasing popularity and demand for DT technology, this tool is expected to play a significant role in the future of robotics and automation across all industries [53].

Integrating Unity with MoveIt provides a powerful development environment for robotics applications. By combining the advanced simulation capabilities of Unity with the powerful motion-planning tools provided by MoveIt using ROS, developers can rapidly develop and test complex robot behaviours in a virtual environment before deploying them on physical hardware. This integration not only accelerates the development process but also enhances the reliability and safety of robotic systems by enabling extensive testing and refinement in a controlled environment. ROS ensures that the DT can not only mirror its physical twin in real time but also adapt and evolve based on continuous data flow [54]. The synergy of Unity’s visualization strengths and ROS’s robotic control capabilities paves the way for advanced DT applications, where real-time feedback and adaptive responses are crucial. The fusion of Unity, ROS, and MoveIt opens a robust development avenue for robotics applications, fortifying the groundwork for a transition towards Industry 5.0. The case study encapsulated in this paper accentuates the essential role of game engines in optimizing operations and bolstering efficiency across industries. This integrated approach also underscores the importance of real-time data processing and adaptive control in modern manufacturing environments.

Latency is a crucial factor as it influences the DT’s responsiveness. In a manufacturing context, low latency ensures that the DT can react in real time to changes in the physical environment, which is vital for applications requiring immediate feedback and adjustment. On the other hand, accuracy is vital for ensuring that the DT accurately mirrors its physical twin. High accuracy in the DT translates to its ability to simulate the physical system and its performance reliably, which is essential for optimizing manufacturing processes and achieving significant improvements in efficiency and quality. Combining low latency and high accuracy in DTs can significantly improve manufacturing practices by enabling more efficient and precise operations. Real-time data synchronization and accurate simulations allow manufacturers to optimize processes, minimize downtime, and enhance product quality [55]. Furthermore, the capability to simulate and test scenarios virtually before physical implementation can conserve time and resources, fostering greater efficiency and innovation in the manufacturing sector [3]. Furthermore, the consistent and reliable performance of the DTs, as evidenced by the accuracy and latency tests, confirms their potential to revolutionize not just manufacturing but also other sectors where robotics plays a critical role. Since DT accurately depicts the physical assets in real time, it can be implemented to respond to unexpected situations or abnormalities during operation, including sensor failures, unforeseen environmental disruptions, and other anomalies that can affect the system’s performance. This can be achieved by continuously monitoring and comparing the expected performance of the physical twin with its actual performance. Thus, by integrating DTs with ML, it is possible to develop automatic self-correcting systems. This exploration paves the way for a more nuanced understanding of the potential harboured by DTs in redefining the landscape of intelligent automation and smart manufacturing.

To achieve the successful integration of DTs into existing workflows, it is necessary to consider socio-technical challenges. One major socio-technical issue is corporate culture, where decisions are often made based on gut feelings rather than data-driven insights. Addressing this requires fostering a culture of data-driven decision-making and ensuring that all stakeholders understand the value and benefits of DT technology. Additionally, political competition among different stakeholders within a company can create resistance to new technologies like DTs [56]. Fostering a culture of innovation with strong leadership support and open communication about the benefits and goals of the new technology can help in alleviating such hindrances [57]. Defining clear strategic goals and objectives to align with DTs’ capabilities with organizational needs and stakeholder expectations is equally important for the same [58]. Another issue is the hype around DTs being presented as a solution to all problems leading to skepticism and distrust within organizations. To navigate this, understanding and managing the hype around DTs with trust-building strategies is crucial for its effective adoption and utilization within organizations [59]. Promoting realistic expectations and providing clear evidence of DTs’ benefits through pilot projects and case studies can help build trust and acceptance. Implementing systematic guidelines and methods for creating DTs is also needed to alleviate hesitancy, particularly among small- and medium-sized enterprises, in adopting DTs [60].

Additionally, comprehensive training programs that cover both technical and the broader implications of changes are crucial to provide to the workforce. These programs can address the concerns of unfamiliarity and uncertainties about adaptation along with closing the skills gap and minimizing resistance [61]. Regulatory compliance, particularly in data security and privacy, is another critical aspect [1,62]. Implementing robust security protocols and conducting regular audits can ensure compliance with relevant regulations and standards. Since DT technology is used in critical systems, organizations must implement a defense-in-depth approach, considering legal, technical, and organizational aspects throughout the DT lifecycle, thereby fostering trust among stakeholders and mitigating potential threats [63]. This comprehensive approach to security and compliance is essential for protecting sensitive data and maintaining the integrity of DT systems.

To promote broad acceptance and implementation, demonstrating the value of DT systems through small-scale pilot projects like ours and sharing success stories can build momentum. Actively engaging stakeholders and developing user-friendly interfaces can help ease the transition. Addressing technical challenges alone is not sufficient; it is also crucial that we tackle socio-technical issues to facilitate the successful adoption and utilization of DT systems by companies. Engaging stakeholders early in the process and maintaining open lines of communication can help in addressing concerns and building trust to ensure that the DT systems meet the needs and expectations of all users.

To thoroughly understand the robustness of the DT system, future research should examine its performance under different operational conditions. While this study offers valuable insights into the latency of the DT system under standard conditions, it is important to recognize that performance metrics may vary under different system loads and network environments. As the size and complexity of the system increase, so does the amount of data that needs to be processed and the computational resources required. This limitation is significant because the real-world deployment of DTs often involves dynamic conditions where the operational demands and network quality can fluctuate. While the current work focuses on assessing DTs under simulated operating conditions, further research in this area would be beneficial for enhancing the reliability and scalability of DT systems, particularly in environments with highly variable operational demands and network conditions. Additionally, the inherent latency of the system, which is partly determined by the underlying communication protocols and hardware configurations, could lead to variations in performance. This aspect was not fully explored in this study and warrants further investigation.

Another limitation of the current study is that the experiments, conducted over an eight-hour period, were performed on a relatively new robotic arm with minimal wear. As the robot undergoes wear and tear over time, it is expected to see wider variations in performance, including potential increases in latency and decreases in accuracy. Future studies should consider long-term testing to capture the effects of mechanical wear on the DT’s performance and reliability. Moreover, the experiments conducted in this study involved the robotic arm operating in isolation, without interfacing with other machines. In real-world manufacturing environments, robots often work in conjunction with other automated systems, such as additional robotic arms. Future research should focus on examining the DT’s performance in such integrated environments, where variations in timing and synchronization become more critical.

Future research should focus on addressing the scalability of DTs by coordinating the robotic arm with other automated systems, such as conveyor belts or additional robotic arms, to simulate a fully integrated smart manufacturing process, as well as improve data processing algorithms to facilitate DT implementation across different industries. Additionally, exploring the human factor in DT interaction, such as developing user-friendly interfaces that facilitate the interaction between operators and DTs, is crucial for ensuring successful deployment.

## 5. Conclusions

The DT presented in this study currently simulates the robotic arm’s joint states, laying the groundwork for a more comprehensive model. Future iterations will aim to incorporate additional parameters, such as speed, acceleration, effort, etc., to enhance the DT’s complexity and realism. By expanding the range of simulated parameters, we can achieve a more detailed and accurate representation of the physical system, enabling better optimization and control. The architecture’s modularity paves the way for seamless integration with other elements within the manufacturing environment, further expanding the DT’s scope. This flexibility ensures that the DT can adapt to various industrial applications, providing a scalable solution for diverse manufacturing needs.

The convergence of this DT with other technologies such as VR, MR, RL, etc. is the way forward to realizing the full potential of DT technology. Exploring the integration of additional sensing modalities and other cutting-edge Industry 4.0/5.0 technologies like Digital Threads and Blockchain can further enrich the DT’s realism and functionality, setting the stage for a comprehensive, modular, and scalable DT architecture adaptable to a myriad of industrial applications. These advancements will not only enhance the DT’s capabilities but also provide new opportunities for innovation and efficiency in manufacturing processes. The modular nature of our proposed architecture bodes well for the incorporation of these advanced technologies, potentially ushering in a new era of intelligent automation underpinned by highly accurate and responsive DTs.

In conclusion, this paper provides a compelling argument for the broader adoption of DTs, particularly those developed using game engines like Unity in conjunction with ROS and MoveIt. The seamless integration of these technologies creates a powerful framework for developing highly detailed and interactive DTs, which are essential for modern smart manufacturing environments. Its role, characterized by the seamless melding of virtual and physical realms, is where continuous innovation is not just a possibility but a necessity. However, in the spirit of Industry 5.0, future enhancements must also consider the human factor. Developing interfaces that are more intuitive and user-friendly will empower workers, offering them a deeper interaction with the DT and the robotic systems it represents. By taking a human-centric approach, we can focus on creating a synergy between the valuable human touch and digital advancements. Overall, the integration of DTs into manufacturing workflows promises to drive significant improvements in productivity, efficiency, and flexibility, setting the stage for the next generation of smart manufacturing solutions.

## Figures and Tables

**Table 1:** Summarized contributions of ROS-based DT developed by researchers.

Engine Used	Key Contributions	Pros	Cons	Reference
Gazebo	Autonomous and remote operations for safety	Manages environmental uncertainties	Limited to specific robot models	[19]
Gazebo	Improved efficiency with multitasking mobile dual-arm robots	Enhances factory productivity	Complexity in integration	[20]
Unity	Trajectory programming and data for ML applications	Suitable for complex environments	High computational requirements	[21]
Gazebo	Safe training environment for students	Cost-effective training	Limited to educational use	[22]
Unreal Engine 4	Real-time monitoring and control for educational purposes	Enhances learning experience	High system requirements	[23]
Unity	Synchronization and advanced user interfaces	Improved teleoperation	Initial setup complexity	[24]
Gazebo	Reconfigurable assembly systems for automotive industry	Facilitates human–robot collaboration	High complexity in dynamic environments	[25]

**Table 2:** ABB IRB 1200 5/0.9 Robot Specifications [42].

Robot Specifications	Axis Max. Speed	Working Range	Range of Motion
Axes: 6			
Payload: 5 kg	Axis 1: 288°/s	Axis 1: +170° to −170°	Axis 1: 340°
Armload: 0.3 kg	Axis 2: 240°/s	Axis 2: +130° to −100°	Axis 2: 230°
Reach: 0.9 m	Axis 3: 300°/s	Axis 3: +70° to −200°	Axis 3: 270°
Position repeatability: 0.025 mm	Axis 4: 400°/s	Axis 4: +270° to −270°	Axis 4: 540°
Robot weight: 54 kg	Axis 5: 405°/s	Axis 5: +130° to −130°	Axis 5: 260°
Robot height: 967 mm	Axis 6: 600°/s	Axis 6: +400° to −400°	Axis 6: 800°
Mounting: Any angle			

**Table 3:** Unity packages used for making digital model and connecting with ROS.

Package	Functionality
ROS-TCP-Connector	Unity package for sending, receiving, and visualizing messages from ROS
ROS TCP-Endpoint	ROS node for sending/receiving messages from Unity
URDF-Importer	Unity package for loading URDF files

**Table 4:** Mean values of actual (MAE) and normalized (MNE) errors.

Joints	Cycle 1		Cycle 2		Cycle 3		Overall	
	MAE	MNE	MAE	MNE	MAE	MNE	MAE	MNE
Joint_1	0.0024	0.0000	0.0022	0.0000	0.0018	0.0000	0.0021	0.0000
Joint_2	0.1633	0.0007	0.1650	0.0007	0.1720	0.0007	0.1666	0.0007
Joint_3	0.0073	0.0000	0.0075	0.0000	0.0071	0.0000	0.0073	0.0000
Joint_4	0.0022	0.0000	0.0022	0.0000	0.0024	0.0000	0.0023	0.0000
Joint_5	0.0005	0.0000	0.0005	0.0000	0.0004	0.0000	0.0005	0.0000
Joint_6	0.0002	0.0000	0.0003	0.0000	0.0002	0.0000	0.0002	0.0000

**Table 5:** Standard deviation of actual (SDAE) and normalized errors (SDNE).

Joints	Cycle 1		Cycle 2		Cycle 3		Overall	
	SDAE	SDNE	SDAE	SDNE	SDAE	SDNE	SDAE	SDNE
Joint_1	0.0206	0.0001	0.0203	0.0001	0.0113	0.0000	0.0181	0.0001
Joint_2	0.1072	0.0005	0.1065	0.0005	0.1066	0.0005	0.1069	0.0005
Joint_3	0.0084	0.0000	0.0148	0.0001	0.0070	0.0000	0.0105	0.0000
Joint_4	0.0144	0.0000	0.0116	0.0000	0.0194	0.0000	0.0154	0.0000
Joint_5	0.0044	0.0000	0.0030	0.0000	0.0030	0.0000	0.0036	0.0000
Joint_6	0.0038	0.0000	0.0024	0.0000	0.0012	0.0000	0.0028	0.0000

## Data Availability

The dataset is available upon request from the authors.
